# Downregulation of programmed cell death 10 is associated with tumor cell proliferation, hyperangiogenesis and peritumoral edema in human glioblastoma

**DOI:** 10.1186/s12885-015-1709-8

**Published:** 2015-10-21

**Authors:** Nicole Lambertz, Nicolai El Hindy, Ilonka Kreitschmann-Andermahr, Klaus Peter Stein, Philipp Dammann, Neriman Oezkan, Oliver Mueller, Ulrich Sure, Yuan Zhu

**Affiliations:** 1 Department of Neurosurgery, Medical Faculty, University of Duisburg-Essen, Hufelandstr. 55, 45147 Essen, Germany; 2 Present Address: Department of Neurosurgery, KRH Klinikum Nordstadt, Haltenhoffstr. 41, 30167 Hannover, Germany

**Keywords:** Primary glioblastoma, PDCD10/CCM3, Tumor cell proliferation, Tumor cell apoptosis, Microvessel density, Angiogenesis, Peritumoral edema

## Abstract

**Background:**

Neovascularization and peritumoral edema are hallmarks of glioblastoma (GBM). Programmed cell death 10 (PDCD10) plays a pivotal role in regulating apoptosis, neoangiogenesis and vessel permeability and is implicated in certain tumor signaling pathways. However, little is known about PDCD10 in GBM. We aimed to investigate the expression pattern of PDCD10 and to identify the association of its expression with some molecular and clinical parameters in human GBM.

**Methods:**

mRNA and protein expression of PDCD10 were examined respectively by real-time RT-PCR and Western blotting in GBM (*n* = 27), astrocytoma grade II (*n* = 13) and control (*n* = 11). The protein level of p-Akt and GFAP was detected by Western blot. Double-imunofluorecent staining was performed to reveal the cellular expression profile of PDCD10. Brain edema and microvascular density (MVD) were respectively analyzed based on pre-operative MRI and after laminin immnostaining. MGMT promoter methylation was detected by methylation specific PCR.

**Results:**

mRNA and protein levels of PDCD10 were significantly downregulated in GBM, concomitantly accompanied by the activation of Akt. PDCD10 immunoreactivity was absent in proliferating tumor cells, endothelial cells and GFAP-positive cells, but exclusively present in the hypoxic pseudopalisading cells which underwent apoptosis. Moreover, loss of PDCD10 was associated with a higher MVD and a more severe peritumoral edema but not with MGMT promoter methylation in GBM.

**Conclusion:**

We report for the first time that PDCD10 expression is downregulated in GBM, which is associated with the activation of Akt signaling protein. PDCD10 is potentially implicated in tumor proliferation and apoptosis, hyperangiogenesis and peritumoral edema in GBM.

## Background

Glioblastoma (GBM) is the most aggressive brain tumor in the central nervous system (CNS) and accounts for approximately 60-70 % of all gliomas. Patient outcome following standard therapy comprised of surgery, radiotherapy, and chemotherapy remains poor. The presence of microvascular hyperplasia and necrotic foci is unique feature of GBM, distinctly distinguished from low grade astrocytoma [1]. Therefore, anti-angiogenic drug regimens are at present increasingly incorporated into combination therapies for GBM [2]. Current anti-angiogenic approaches mainly include anti-VEGF pathway and anti-VEGF-independent pathways. These approaches have clearly improved the response to radiological therapy and reduced brain edema, but do not significantly influence overall survival of GBM patients. Thus, to bridge this gap, the identification of emerging molecular targets for GBM therapy becomes increasingly important.

Programmed cell death 10 *(PDCD10)* has originally been referred to as TFAR15 (TF-1 cell apoptosis related gene) and is also known as cerebral cavernous malformation 3 (CCM3). Mutations of *PDCD10* predispose to the development of human familial cerebral cavernous malformations (CCM), a brain vascular anomaly involving aberrant angiogenesis and chronic hemorrhage [3, 4]. *PDCD10* is evolutionary conserved and is ubiquitously expressed in nearly all tissues. In the CNS, PDCD10 is expressed in neuronal, glial and endothelial cells [5, 6]. Biochemically, PDCD10 is an adaptor protein that can bind to different proteins and/or protein complexes such as CCM2; each of three germinal center kinases III (GCKIII) serine/threonine kinases (STK24, STK25 and MST4); paxillin and VEGFR2 [7]. The crucial role of PDCD10 in vascularization and in angiogenesis has been well documented [8–11]. Moreover, much attention has recently been drawn to the study of the PDCD10 function in vessel permeability due to the aggressive hemorrhagic behavior observed in cerebral cavernous malformation patients harboring a *PDCD10* mutation [12] and after loss of heterozygosity (LOH) for *Pdcd10 in* mice [8]. Loss of PDCD10 leads to the disruption of endothelial cell-cell junctions, to impairment of vascular stability through hyperactivation of RhoA and to an increase in stress fiber assembly [13]. In addition to its established endothelial function, PDCD10 is also essential for the neuron-glial unit. Vascular pathology has been induced after targeted deletion of *Pdcd10*/*Ccm3* in murine neuroglia [14] and PDCD10 has been found to be required for the neuronal migration [15].

Increasing evidence indicates a pivotal role of PDCD10 in regulating cell survival and death. Both anti-apoptotic [16–18] and pro-apoptotic functions of PDCD10 [19–22] have been reported in different type of cells, suggesting a context-dependent apoptotic function of PDCD10. Moreover, gene chip analysis indicated the involvement of PDCD10 in tumor signaling [23] and in resistance to chemotherapy-triggered apoptosis [24].

The signaling pathways underlying the angiogenesis, vascular permeability and apoptotic functions of PDCD10 have been intensively studied and reviewed in recent publications [7, 25]. In our group, we have reported that silencing *PDCD10/CCM3* stimulated endothelial angiogenesis through activating VEGF signaling and impairing Dll4-Notch signaling. Indeed, loss of PDCD10 induced apoptosis resistance in endothelial cells after apoptotic stimuli, accompanied by the activation of p38, Erk1/2, and Akt signaling proteins [22, 26].

In line with the crucial role of PDCD10 in angiogenesis, vessel permeability and apoptosis and based on the altered expression of *PDCD10* in various cancers, we assumed that PDCD10 could be potentially involved in the pathology of GBM. To this end, we studied the expression of PDCD10 at both mRNA and protein levels, and characterized the regional and cellular expression profile of this protein in GBM. The expression of PDCD10 was correlated to the tumor cell survival signaling protein p-Akt, to microvascular density and peritumour edema in GBM.

## Methods

### Patient cohort and magnetic resonance imaging (MRI)-based edema grading

The study comprised 27 primary GBM and 13 astrocytoma WHO grade II (Astro II) adult patients, respectively, who underwent surgery from 2009 to 2011 at our department. All experiments were performed with histopathologically confirmed tumor material containing the tumor core and the infiltration zone. The surgical specimens of patients who underwent anterior temporal lobe resections due to temporal lobe epilepsy were used as control tissue (*n* = 11). Preoperative MRI was used to grade peritumoral edema in all patients according to the protocols established in our previous study [27]. Briefly, grading was performed only if edema was detected in the preoperative image; otherwise the patient was included in the “grade 0” subgroup. Edema was identified as a peritumoral hyperintense signal in T2-weighted MRI. Patients were categorized as grade 1 if the edematous area was less than tumor volume, as grade 2 if the edematous and tumor volumes were equal and as grade 3 if the edematous area was larger than the tumor mass. Brain edema grading was performed prior to determining PDCD10 expression by two independent investigators. The study was strictly performed according to the Declaration of Helsinki and approved by the local ethics committee of the University Hospital of Essen. Informed consent was obtained from the patients.

### Real-time reverse transcription-PCR (RT^2^-PCR)

Total RNA and cDNA synthesis were performed as described in our previous publication [27]. The primers and the real time-PCR settings were used according to the established protocol [26]. Relative mRNA expression (fold of change) was calculated according to the cycle threshold approach (2^-∆∆Ct^ method) and normalized to the reference gene *GAPDH*.

### Western blot

Total protein extraction and electrophoresis were done according to the previous protocol [27]. The following primary antibodies were used: rabbit anti-PDCD10 (Atlas; 1:400), rabbit anti-GAPDH (14C10) (Cell Signaling; 1:1000), rabbit anti-phospho-Akt (p-Akt, Cell Signaling; 1:600), rabbit anti-RhoA (Santa Cruz Technology, 1:200), rabbit anti-phospho-myosin light chain 2 (Thr18/Ser19) (p-MLC2) (Cell Signaling; 1:1000) and mouse anti-GFAP (Sigma Aldrich; 1:5000), For semi-quantification of the blot, integrated optical density (IOD) of the bands was measured by Image J software. The relative expression of a target protein was calculated by the IOD ratio of the target protein to the housekeeping protein GAPDH, and the data were presented as percentage of the control.

### Immunostaining

Immunostaining of laminin was performed as described previously [27]. For immunofluorescent staining of PDCD10, sections were incubated with rabbit anti-PDCD10 (Atlas, 1:65) at 4 °C overnight and then incubated with biotinylated goat anti-rabbit IgG (Dako, 1:400) at 37 °C for 1 h followed by the substrate reaction with FITC-labelled avidin (Dako, 1:400) at room temperature for 1 h. For double-immunofluorecent staining, the following antibody mixtures were applied to the sections: rabbit anti-PDCD10 (Atlas, 1:65) and mouse anti-GFAP (Sigma, 1:200); rabbit anti-PDCD10 and mouse anti-CD31 (Dako, 1:20); rabbit anti-PDCD10 and mouse anti-CD68 (Dako, 1:100); rabbit anti-PDCD10 and mouse anti-PCNA (Dako, 1:200); mouse anti-PDCD10 (Santa Cruze, 1:50) and rabbit anti-caspase 3 (active form) (Cell signaling, 1:400). After incubation overnight, the sections were incubated with the mixture of biotinylated goat anti-rabbit IgG (1:400) and Texas red anti-mouse IgG (H + L) (Vector Laboratories, 1:200) followed by the substrate reaction with FITC-labelled avidin. Counterstaining was performed with Hoechst-33258. The sections were finally analyzed by using a fluorescence microscope with ApoTome System (Zeiss, Axio Imager M2).

### Evaluation of microvascular density (MVD) and O6-methylguanine-methyltransferase (MGMT) promoter methylation

MVD evaluation and methylation specific PCR (MSP) analysis of *MGMT* promoter methylation were performed using the protocols established in our previous study [27].

### Statistical analysis

All statistical analyses were performed using the Graph-Pad-Prism Software Version 4. Student’s *t* test with Welch’s correction was performed for data analysis. *P* < 0.05 was considered as significant difference between groups.

## Results

### Downregulation of PDCD10 expression in GBM

**Fig. 1:**
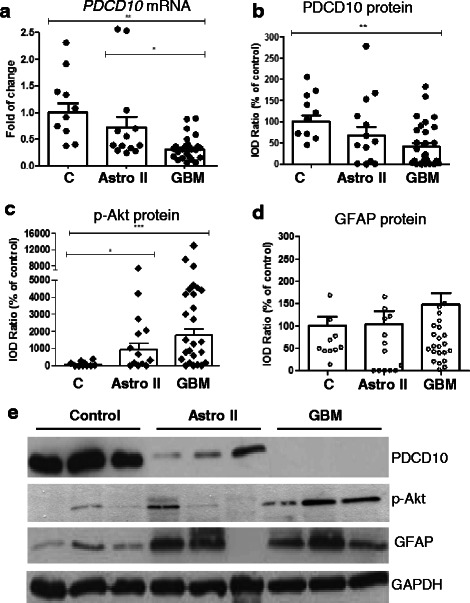
Downregulation of PDCD10 was correlated to the activation of Akt in GBM. **a** Downregulation of *PDCD10* mRNA. Real time RT-PCR demonstrated a downregulation of PDCD10 in a malignancy dependent manner in glioma. **b**-**d** Downregulation of PDCD10 protein expression was inversely correlated to the level of p-Akt in GBM. Semi-quantification of the blots confirmed a significant downregulation of PDCD10 protein level in GBM (**b**), which was inversely correlated to an activation of Akt (**c**). The level of GFAP was not significantly different among the control (**c**), astrocytoma grade II (Astro II) and GBM (**d**) group. **e** A representative blot showed the expression of PDCD10, phosphor-Akt (p-Akt), and GFAP in control (**c**), Astro II and GBM. * *p* < 0.05, ** *p* < 0.01 and *** *p* < 0.001, compared to **c**

RT^2^-PCR revealed a significant downregulation of *PDCD10* in GBM compared to the control (*p* < 0.01) and to the Astro II group (*p* < 0.05). Of note, 23 out of 27 GBM samples (85.2 %) exhibited individual mRNA levels below the mean value of “fold of change”, indicating a dominant downregulation of this gene in the majority of investigated GBM cases (Fig. 1a). Western blot confirmed an approximately 60 % reduced expression of PDCD10 in GBM in comparison to the control group (Fig. 1b) (*p* < 0.01). The expression of mRNA and protein of PDCD10 in Astro II was slightly decreased but this was not statistically different from that in controls. Interestingly, the level of p-Akt, a crucial signaling protein for tumor cell survival and proliferation, was inversely associated with PDCD10 expression in Astro II (*p* < 0.05) and in GBM (*p* < 0.001) (Fig. 1c), whereas GFAP, a marker of astrocytoma tumors and expressed in differentiated astrocytes, was detected at varying levels in individual tumor tissues and did not show a significant difference among three groups (Fig. 1d and e).

### PDCD10 immunoreactivity was absent in the majority of endothelial and tumor cells but was exclusively detected in pseudopalisades of GBM

**Fig. 2:**
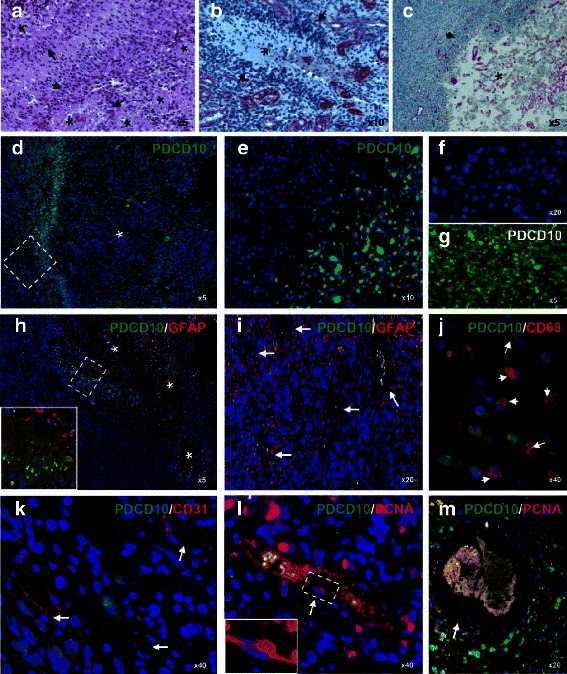
Characterization of the regional and cellular localization of PDCD10 immunoreactivity in GBM. **a** Histopathological feature of GBM. H&E staining outlined multiple pseudopalisades. A typical pseudopalisade is composed of peripheral cellular pseudopalisading (*arrows*) around a necrotic center (*asterisks*). **b** and **c** Laminin staining. Microvascular hyperplasia (*violet-colored structure*) was found predominantly close to the pseudopalisading. Peripheral cellular pseudopalisading (*arrows*) was highlighted by the counterstaining. **d**-**g** Immunostaining of PDCD10. PDCD10 immunoreactivity was absent in the necrotic center (*asterisk* in **d**). High magnification view of the white box in **d** indicated that PDCD10 immunoreactivity was not detected in infiltrating tumor cells distant from necrotic area but was exclusively present in peripheral cellular pseudopalisading (**e**). Negative staining control omitting the primary antibody did not show any detectable signal (**f**), whereas the staining on a control brain section detected intensive immunoreactivity of PDCD10 (**g**). **h**-**m** Double staining of PDCD10 (*green*) with different cellular markers (*red*). PDCD10-positive cells in peripheral cellular pseudopalisading were not co-localized with GFAP (low magnification view in **h** and high magnification view in the inserted box). In the necrosis-distant area (infiltration area), tumor cells were positively labelled with GFAP but were negative to PDCD10-staining (**i**); meanwhile numerous microvessel-like structures in this infiltration area did not show PDCD10 immunoreactivity (*arrows* in **i**). Absence of PDCD10 in endothelial cells of the microvessels was confirmed by the double staining of PDCD10 and CD31 (*arrows* in **k**). These PDCD10-negative vessels exhibited proliferating activity as evidenced by the staining with PCNA (*arrow* and inserted *box* in **l**). In contrast, the thrombosed vessel (*arrow* in **m**) did not label with PCNA rather induced PDCD10 expression in surrounding tumor cells (**m**). Some of PDCD10 positive cells were co-labelled with macrophage marker CD68 (*arrows* in **j**)

Microscopically, necrosis and microvascular proliferation are typical morphologic features of GBM which distinguish this tumor from low grade astrocytoma. As shown in Fig. 2a-c, tumor cells in GBM were often accumulated and arranged in a perpendicular (pseudopalisading) fashion (arrows) around necrotic areas (asterisks). The necrotic center was either built by a clear zone (less sever necrosis) (asterisk in Fig. 2b) or by severely necrotic cells (asterisk in Fig. 2c). Microvascular hyperplasia as shown by laminin staining (violet-colored vessels in Fig. 2b and c) was often found in adjacent pseudopalisading area where tumor cell growth was sustained.

Next, we focused on identifying the regional and cellular expression profile of PDCD10 in GBM by immunofluorecent staining of PDCD10 on GBM sections. No immune-signal was detected in negative staining control in which non-specific Ig, instead of primary antibody, was applied to the section followed by count-staining with Hoechst 33258 (Fig. 2f). In contrast, intensive immunoreactivity of PDCD10 was observed on a control brain section (Fig. 2g). In GBM sections, PDCD10 immunoreactivity was absent in the central clear zone (asterisk in Fig. 2d) and in the tumor cell dense infiltration area. Surprisingly, PDCD10 was exclusively detected in the peripheral cellular pseudopalisades as shown in Fig. 2d. Figure 2e is the high magnification view of the dash-lined area of Fig 2d. This distinct PDCD10 expression manner was also observed in the pseudopalisades with a more pronounced necrotic center (Fig. 2h) and in throughout GBM sections. Double staining of PDCD10 and GFAP revealed no overlapped expression of PDCD10 and GFAP in pseudopalisading cells (Fig. 2h) and in the infiltration zone (Fig. 2i), suggesting that PDCD10 expression is independent of astrocytic differentiation. It is noteworthy that the numerous microvessel-like structures in the infiltration zone exhibiting PDCD10-negative staining (arrows in Fig. 2i). Double staining of PDCD10 and the endothelial marker CD31 confirmed this observation (Fig. 2k), and furthermore, these PDCD10-negative microvessels showed proliferation activity as evidenced by the double staining of PDCD10 (green) and PCNA (red) (Fig. 2l). As shown in a high magnification view (inserted white box in Fig. 2l) of dash-lined area in Fig. 2l, PCNA immunoreactivity was clearly detected in the nucleus of a PDCD10-negative endothelial cell. Interestingly, the thrombosed microvessels in/nearby the necrotic area were PCNA negative (arrow in Fig. 2m) but often accompanied by the induction of PDCD10 expression in the area surrounding the thrombosed vessel (Fig. 2m), suggesting a hypoxia-induced PDCD10 expression. Some of these PDCD10-expressing cells were positively labelled with CD68 (arrows in Fig. 2j), indicating PDCD10 expression in macrophage.

### PDCD10 expression was inversely correlated to the proliferation of tumor cells and vascular endothelial cells but was positively associated with apoptosis

**Fig. 3:**
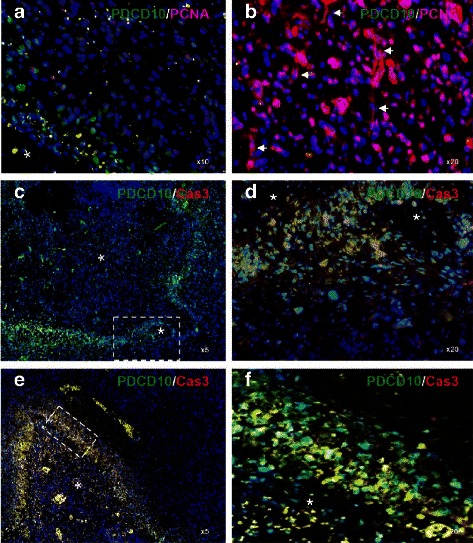
The association of PDCD10 expression with tumor cell proliferation and with caspase 3 activation. **a** and **b** Double staining of PDCD10 and PCNA in GBM. PDCD10-positive cells (*green*) exclusively detected in pseudopalisading did not co-stain with PCNA (*red*) (**a**), whereas nearly all tumor cells the tumor microvessels (*arrows* in **b**) in the infiltration area appeared negative to PDCD10 staining but showed strong PCNA immunoreactivity (*red*), indicating an inverse correlation of PDCD10 expression and proliferation status of tumor cells and microvessels (**b**). **c**-**f** Double staining of PDCD10 and active caspase 3. Co-localization of the immunoreactivity of PDCD10 (*green*) and active caspase 3 (Cas3) (*red*) was exclusively detected in peripheral cellular pseudopalisadings, suggesting that an association of PDCD10 expression and apoptosis. Of note, the selective expression of PDCD10 in the pseudopalisading area was independent of whether pseudopalisading formed around a slight (**c**) or a severe necrotic zone (**e**). **d** and **f**, respectively, show the high magnification view of the white boxes in **c** and **d**. Asterisk in **a** and **c**-**f** indicates the necrotic center

To further investigate the association of PDCD10 expression and the status of tumor cells, i.e., proliferation or apoptosis, we performed double staining of PDCD10/PCNA and PDCD10/caspase 3 (active form). In pseudopalisades, PDCD10-positive cells (green) did not co-stain with PCNA (Fig. 3a) but were exclusively co-labelled with active caspase 3 as revealed in low (Fig. 3c) and high (Fig. 3d) magnification views of the GBM section. The co-expression of PDCD10 and active caspase 3 was also detected in the pseudopalisades with a severely necrotic center (Fig. 3e and f). In contrast, nearly all tumor cells and microvessel endothelial cells (arrows in Fig. 3b) in the infiltrating zone were absent of PDCD10 immuno-signal (green) but showed strong PCNA immunoreactivity (red) (Fig. 3b) and appeared negative for caspase 3 staining (Fig. 3c and e).

### The association of PDCD10 expression with microvascular density (MVD)

**Fig. 4:**
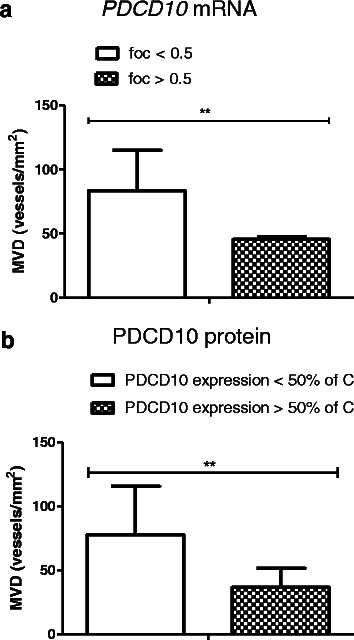
Inverse association of the expression of PDCD10 with microvascular density (MVD) in GBM. **a** The association of PDCD10 mRNA expression and MVD. When GBM was subgrouped based on the mRNA level of PDCD10 (“fold of change” (foc) < 0.5, *n* = 20; or > 0.5, *n* = 7), a significantly higher MVD was observed in the subgroup expressing lower PDCD10 (foc < 0.5) in GBM. **b** The association of PDCD10 protein expression and MVD. When GBM was subgrouped based on the protein level of PDCD10 < 50 % of control (*n* = 22); or > 50 % of control (*n* = 5), a significantly higher MVD was observed in the subgroup expressing PDCD10 < 50 % of control. ** *p* < 0.01, compared to the group expressing lower mRNA (**a**) or protein (**b**) of PDCD10

MVD is a parameter associated with angiogenesis in solid tumors. When GBM patients were subgrouped based on the *PDCD10* mRNA level, MVD was 37 vessel/mm^2^ and 78 vessel/mm^2^ in the subgroups of “fold of change (foc) > 0.5” and “foc < 0.5”, respectively (*p* < 0.01) (Fig. 4a). Similarly, MVD was significantly higher in the GBM subgroup expressing lower PDCD10 protein (<50 % of the control) than that with higher PDCD10 expression (> 50 % of the control) (*p* < 0.01) (Fig. 4b). These results indicate an inverse correlation of PDCD10 expression and MVD in GBM.

### Association of PDCD10 expression with methylation status of MGMT and with peritumoral edema

Hypermethylation of the MGMT gene has been shown to be associated with improved outcome in glioblastoma (GBM) and may be a predictive marker of sensitivity to chemotherapy with alkylating agents and to radiation therapy in GBM. Thus, the promotor methylation status of MGMT is routinely examined in GBM patients in our hospital and most other tertiary care centers. Among 27 GBM, 10 of 27 GBM (37 %) were positive for the promoter methylation of MGMT. The association analysis revealed that the mean of “fold of change” of *PDCD10* mRNA was 0.30 ± 0.08 and 0.42 ± 0.07 in MGMT-methylated GBM and in MGMT-non methylated cases, respectively (*p* > 0.05), suggesting that MGMT methylation status is not associated with PDCD10 expression level in GBM.

**Fig. 5:**
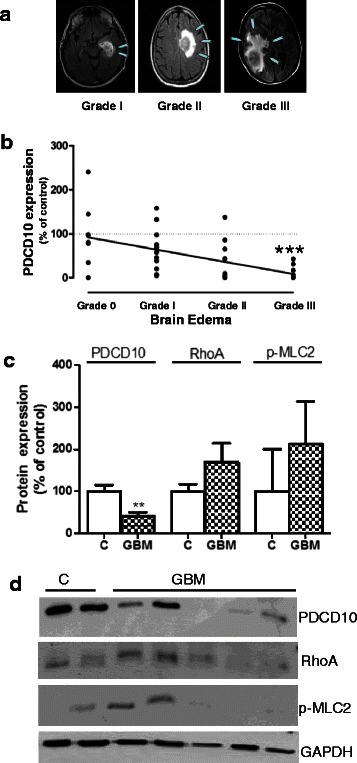
The association of PDCD10 expression and the grade of brain edema in GBM. **a** Grading peritumoral edema in GBM. The grade of peritumoral edema in GBM was evaluated based on magnetic resonance imaging (MRI). **b** The association of PDCD10 protein expression and the grade of edema. The expression of PDCD10 was inversely correlated to the grade of peritumoral edema. *** *p* < 0.001, compared to edema grade 0. **c** and **d** The expression of total RhoA and phosphor-MLC2 (p-MLC2) in GBM. The expression of RhoA and phosphor-MLC2 (p-MLC2) appeared to be increased in GBM compared to the control (**c**) but did not show statistically significant difference (**c**). Thus, downregulation of PDCD10 did not seem to be correlated to the activation of RhoA signaling. The representative blots are shown in **d**. ** *p* < 0.01, compared with control (**c**)

PDCD10 has been shown to affect vessel permeability through RhoA signaling [13, 14, 28, 29]. We thus evaluated peritumoral edema and analyzed the association of the grade of edema with PDCD10 protein expression and with p-MLC2, a downstream kinase of RhoA. Figure 5a is representative of different edema grades in GBMs. Interestingly, the PDCD10 protein expression decreased in an edema grade-dependent manner in GBM (Fig. 5b). The patients with a severe peritumoral edema (grade III) expressed a significant lower protein level of PDCD10 in comparison to the cases without edema (grade 0) (*p* < 0.001) (Fig. 5b). The expression of p-MLC2 seems to be correlated to the level of total RhoA in GBM, but both p-MLC2 and RhoA levels appeared varying in individual GBM tissues (Fig. 5d). Semi-quantification of p-MLC2 and RhoA blots did not reveal statistically significant difference between GBM and the control group (Fig. 5c).

## Discussion

In contrast to the well-established function of PDCD10 in vasculogenesis, neo-angiogenesis, vessel remodeling and vessel permeability, only limited information is available on PDCD10 in malignant tumors despite its original description as an apoptosis related gene [30]. Here we report for the first time that PDCD10 expression at both mRNA and protein levels decreased in a malignancy-dependent manner in glioma. A significant downregulation of PDCD10 was found in GBM, the most aggressive glioma, concomitantly accompanied by the activation of Akt. Loss of PDCD10 immunoreactivity was identified in proliferating tumor and vascular endothelial cells as well as in tumor cells. Interestingly, PDCD10 immunoreactivity was selectively detected in pseudopalisading cells which were positively labelled with active caspase 3, suggesting a possible direct role of PDCD10 in regulating apoptosis in this hypoxia region. Moreover, downregulation of PDCD10 was associated with a higher microvascular density, and with more severe peritumoral edema in GBM. These results indicate that PDCD10 expression is deregulated in GBM, which is potentially involved in the regulation of tumor cell proliferation and apoptosis and in the pathology of neo-angiogenesis and peritumoral edema in GBM.

### Distinct PDCD10 expression in GBM: Lack of PDCD10 expression in microvessels and in infiltrating tumor cells but selective expression in pseudopalisading cells

PDCD10 is highly conserved across species and is ubiquitously expressed in all normal tissues including brain tissue. Aberrant expression of *PDCD10* gene transcription has previously been detected in various types of human cancers [31]. Indeed, an altered expression of *PDCD10* was found in response to different chemotherapies, giving rise to the hypothesis that PDCD10 is involved in chemoresistance in certain cancers [24, 32]. However, it is unclear whether the expression of this apoptosis related gene/protein is altered in GBM. Here, we reported for the first time the downregulation of PDCD10 mRNA and protein in a malignancy-dependent manner in gliomas, accompanied by a significant activation of Akt, an important signal protein regulating cell survival and angiogenesis (Fig. 1). To date, only few reports have regarded the regulation mechanism of PDCD10 expression. Chen et al., identified c-Myc as an important coordinator for the transcription of *PDCD10* [33]. Transcription of *PDCD10* was driven by the binding of c-Myc to a non-canonical E-Box element in the promoter of *PDCD10*; and moreover, this binding can be interfered by the methylation at a specific nucleotide in this E-Box thereby suppressing the transcription of *PDCD10*. Whether this mechanism accounts for the downregulation of PDCD10 detected in GBM needs to be further studied in the future.

The histopathologic features that distinguish GBM from lower grade astrocytoma include the presence of pseudopalisading necrosis and microvascular hyperplasia (Fig. 2a-c). The former consists of necrosis foci, usually surrounding cellular pseudopalisades; and the latter is a form of neo-angiogenesis. These two characteristics are thought to contribute mechanically to the transition of lower grade of astrocytoma to GBM and are the most powerful predictors of poor prognosis in GBM [34]. This unique pathological feature of GBM seems to be the consequence of hypoxia. Brat DJ et al., found that more than 50 % of pseudopalisades contained degenerated or thrombosed vascular lumina [34], which leads to regional hypoxia and the outward migration of glioma cells away from the hypoxia area. The majority pseudopalisading cells undergo apoptosis but not proliferation [35]. The present study identified the absence of PDCD10 expression in tumor cells and endothelial cells in the infiltration area and in the necrotic center of GBM but an exclusive expression of PDCD10 in some pseudopalisading cells, indicating a distinct regional expression manner of PDCD10 (Fig. 2d and e; h and i). We assume that the expression of PDCD10 in pseudopalisading cells results from hypoxia because upregulation of PDCD10 was observed in response to oxidative stress [21] and to hypoxia stimuli (own unpublished data). This hypothesis could be also supported by the detection of PDCD10 immunoreactivity in the area nearby the thrombosed and non-proliferating vessel (Fig. 2m).

### The association of PDCD10 expression with cell proliferation and apoptosis

By means of double staining, we further characterized the cellular location of PDCD10 immunoreactivity and the potential association of its expression with cell proliferation and apoptosis. Lack of PDCD10 in CD31- and PCNA-positively vascular endothelial cells (arrows in Fig. 2k and l and in Fig. 3b) suggests that loss of PDCD10 is associated with endothelial proliferation. This observation is in agreement with our previously finding that silencing *PDCD10* in endothelial cells stimulated endothelial angiogenesis including proliferation, migration, sprouting and angiogenesis [22, 26]. Furthermore, we demonstrated that loss of PDCD10 immunoreactivity in tumor cells in the infiltration area was associated with tumor cell proliferation (Fig. 3b), whereas PDCD10-expression cells in pseudopalisades were co-labelled with active caspase 3 (Fig. 3c-f). These results indicate an inverse association of PDCD10 expression with the tumor cell proliferation but a positive correlation with apoptosis, suggesting an implication of PDCD10 in determining the fate of tumor cells in GBM.

### Downregulation of PDCD10 was inversely associated with the microvascular density and with the grade of peritumoral edema

Massive neo-angiogenesis and peritumoral edema are hallmarks of GBM. Growth of malignant gliomas is at least partially dependent on neo-angiogenesis, whereas vasogenic brain edema is a direct consequence of the vascular abnormalities in GBM and is a significant cause of morbidity of GBM [36]. In spite of its well-established pro-angiogenic function [22, 26] and vessel hyper-permeability [13, 14, 28, 29] induced by PDCD10-deficiency, the present study reported for the first time that the downregulation of PDCD10 is inversely associated with MVD (Fig. 4) and with a higher grade of brain edema in GBM (Fig. 5). It has been previously shown that loss of PDCD10 leads to the disruption of endothelial cell-cell junctions and to impair vascular stability through hyperactivation of RhoA and an increase in stress fiber assembly [13]. However, Chan et al. showed that silence of PDCD10 in endothelial cells did not affect RhoA signaling but resulted in lumen formation defect [8]. In the present study, we did not observe a significant increase in p-MLC2 expression in GBM, suggesting no correlation between PDCD10 downregulation and activation of RhoA signaling. Therefore, it is an issue of future interest to study the underlying signaling pathway of PDCD10 mediating peritumoral edema in GBM.

Heterozygous loss-of-function mutations in *PDCD10/CCM3* may predispose to familial CCM [3, 4, 12]. The underlying mechanism of PDCD10 in the pathogenesis of CCM has been extensively studied [7, 25, 37]. Interestingly, the clinical relevance of CCM and brain tumors has been observed by independent groups and reviewed by Mian MK et al [38], although the co-incidence of CCM and brain tumors is rare. Several studies have shown that a low grade astrocytoma [39], or GBM [38, 40] or an intracranial ependymoma [41] occurred at the site of a pre-exiting CCM lesion, and importantly these patients often had a CCM family history. A recent study has highlighted the possible association of CCM with meningiomas in a large cohort of familial CCM harboring *PDCD10* mutations [12]. However, a causal relationship of CCM and other brain tumor remains unproven. Nevertheless, these data suggest a potential genetic mechanism implicated in the collision of CCM and intracranial neoplasms.

## Conclusion

PDCD10 is a remarkably conserved and ubiquitously expressed protein. Significant downregulation of this protein in GBM implies its possible involvement in the pathology of GBM. The association of PDCD10 downregulation with the activation of Akt, with hyper-angiogenesis and with higher a grade of peritumoral edema suggests a potential function of PDCD10 in tumor cell survival, angiogenesis and in regulating vessel integrity. The absence of PDCD10 immunoreactivity in proliferating tumor and its concomitant co-localization with apoptotic marker in pseudopalisade tumor cells may suggest the involvement of *PDCD10* in the progression of GBM. Further investigation is required to uncover the direct role and the underlying signaling of PDCD10 deficiency in GBM, which will extend our understanding on the function of PDCD10 in this tumor.

## Abbreviations

CCM: Cerebral cavernous malformation
CCM3: Cerebral cavernous malformation 3
CNS: Central nervous system
foc: Fold of change
GBM: Glioblastoma
GCKIII: Germinal center kinase III
IOD: Integrated optical density
MGMT: O6-methylguanine-methyltransferase
MRI: Magnetic resonance imaging
MSP: Methylation specific PCR
MVD: Microvascular density
p-Akt: phospho-Akt
p-MLC2: phospho-myosin light chain 2 (Thr18/Ser19)
PDCD10: Programmed cell death 10
RT^2^-PCR: Real time reverse transcription-PCR
TFAR15: TF-1 cell apoptosis related gene
